# Direct Measurement of the Radiative Pattern of Bright and Dark Excitons and Exciton Complexes in Encapsulated Tungsten Diselenide

**DOI:** 10.1038/s41598-020-64838-z

**Published:** 2020-05-15

**Authors:** Lorenz Maximilian Schneider, Shanece S. Esdaille, Daniel A. Rhodes, Katayun Barmak, James C. Hone, Arash Rahimi-Iman

**Affiliations:** 1 Faculty of Physics and Materials Sciences Center, Philipps-Universität Marburg, Marburg, 35032 Germany; 2 Department of Mechanical Engineering, Columbia University, New York, NY 10027 USA; 3 Department of Applied Physics and Applied Mathematics, Columbia University, New York, NY 10027 USA

**Keywords:** Two-dimensional materials, Fluorescence spectroscopy, Two-dimensional materials

## Abstract

The optical properties of particularly the tungsten-based transition-metal dichalcogenides are strongly influenced by the presence of dark excitons. Recently, theoretical predictions as well as indirect experimental insights have shown that two different dark excitons exist within the light cone. While one is completely dark, the other one is only dipole forbidden out-of-plane, hence referred to as grey exciton. Here, we present angle-resolved spectroscopic data of a high-quality hexagonal-BN-encapsulated WSe_2_ monolayer with which we directly obtain the radiation pattern of this grey exciton that deviates from that of the bright exciton and other exciton complexes obtained at cryogenic temperatures.

## Introduction

Transition-metal dichalcogenides (TMDCs) have recently drawn lots of attention due to their extraordinary rich exciton physics^1^, showing trions^2,3^, biexcitons^4^ and other higher-order exciton complexes^5,6^. Furthermore, caused by their huge binding energy, higher-order states of the Rydberg-like series can be even seen at room temperature^7,8^. In addition, even polaritons^9^ and valley polaritons^10,11^ can be observed in suitable microcavity structures at these temperatures. With recent publications, it has been revealed that several dark excitons^12,13^ exist that strongly affect the dynamics^14,15^ and spectral features^13,16^ of the system. While there have been several experimental studies for the dark exciton^17–20^, little has been reported about their emission characteristics.

**Figure 1:**
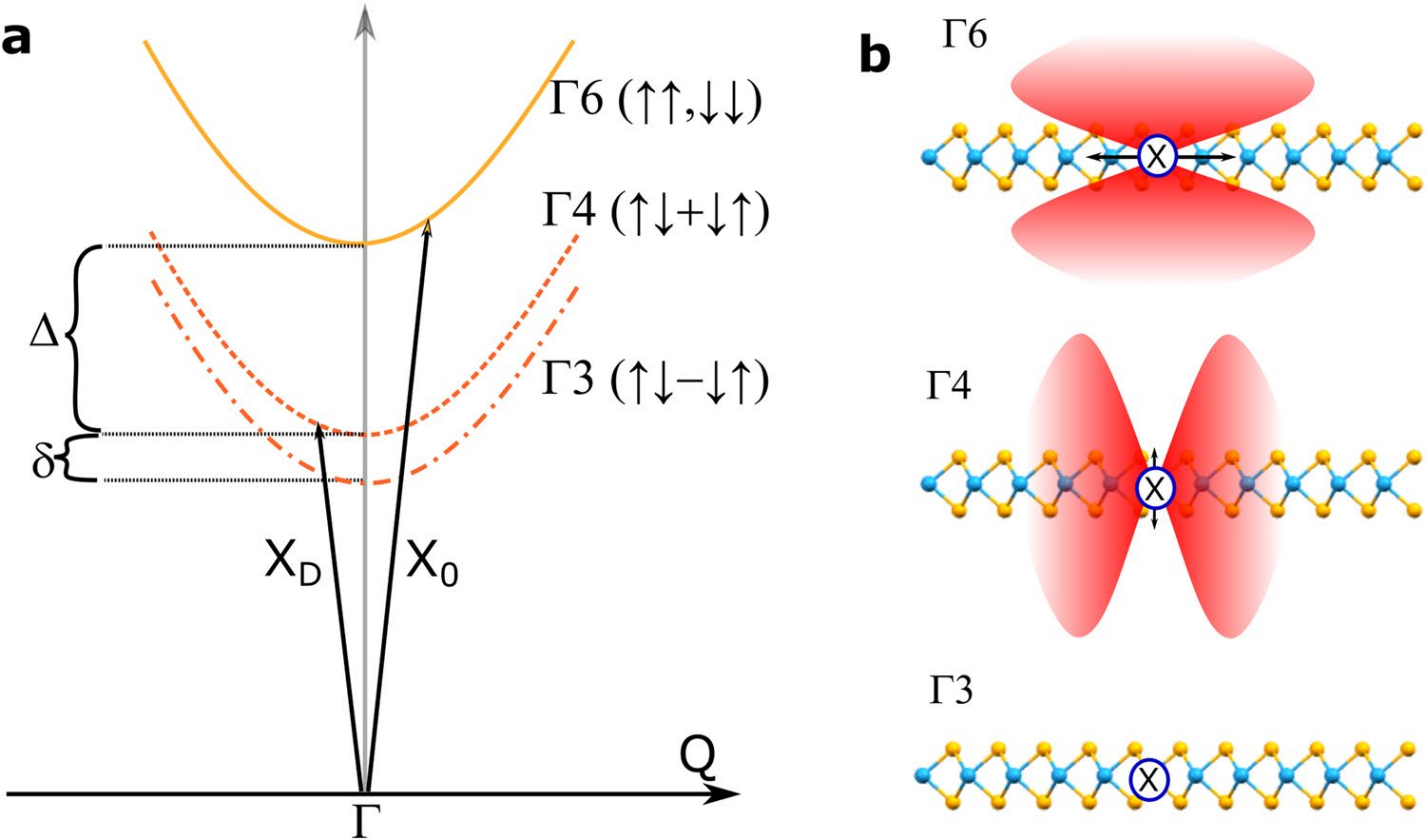
Schematic drawing of the excitonic non-degeneracy and the dark exciton landscape in tungsten diselenide. (**a**) Possible bright and dark exciton states for neutral WSe_2_ arising from the crystal symmetry, indicated in the two-particle picture (energy *E* vs. centre-of-mass momentum *Q*) around zero momentum (Γ point). The bright exciton (X_0_ – Γ6) is separated from the grey exciton (X_D,g_ – Γ4) and the dark exciton (X_D_ – Γ3). Arrows indicate the optical excitation of the respective states. In contrast to bright excitons, the grey exciton is only dipole allowed for z-polarization (corresponding to an out-of-plane dipole). A sketch of the expected radiation pattern for the excitons with different symmetries is shown in (**b**).

Recent detailed group-theory analysis of the possible excitons’ branches^15^ of WSe_2_ has shown that four different exciton configurations exist within the light cone at the A-exciton peak (cf. Figure 1). Firstly, two bright excitons (Γ6) at K respectively K’ are provided, where hole and electron exhibit the same spin. Secondly, owing to a small but nonnegligible intravalley interaction, two more possible states arise that represent a coherent superposition of intervalley excitons composed of the spin-forbidden transition across K and K’ valley (irreducible representations Γ4 and Γ3). A schematic two-particle picture for these species is shown in Fig. 1a, whereas their radiation pattern is indicated in Fig. 1b. The Γ4 state transforms like a z-component of a vector of D_3h_ group, rendering it a dipole-allowed transition for z-polarization (corresponds to the direction out of plane, as indicated by small arrows attached to the exciton representation in Fig. 1b). In contrast, the Γ3 state is completely dark, i.e. it cannot couple to the electromagnetic field.

This bears huge implications, as the Γ4 can only emit in the plane and not out of the plane. The energetic difference between bright and grey exciton will be here labelled Δ, whereas the one between grey and dark exciton will be represented by δ (cf. Fig. 1a).

This implication has already been investigated using a micro-photoluminescence (µ-PL) setup with separate detection paths for optical x- and z- polarisation^17^ for encapsulated samples. For a bare substrate-supported monolayer at room temperature, the radiation pattern of emission has also been addressed by Fourier-space spectroscopy^21^. However, to the best of our knowledge, no direct measurement of the radiation pattern has been performed for the dark exciton. Here, we present an angle-resolved photoluminescence (PL) study with the focus on the distinct radiation patterns of bright and dark states.

## Results

**Figure 2:**
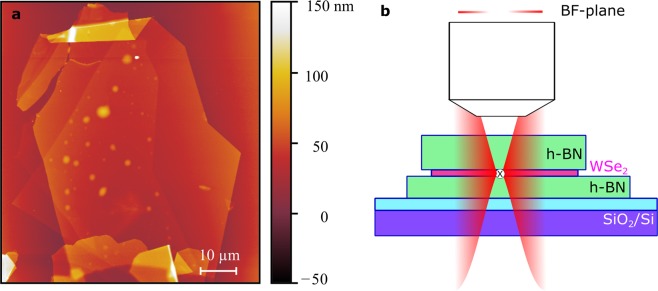
Sample image and measurement concept. (**a**) AFM measurement of the sample and (**b**) conceptual drawing of the measurement showing a schematic radiation pattern for an in-plane radiation from the exciton and the resulting expected intensity distribution in the back-focal (BF) plane of the microscope objective.

The angle-resolved photoluminescence measurement has been carried out on an h-BN encapsulated WSe_2_ monolayer. An atomic-force microscopy (AFM) image of the assembled stack is shown in Fig. 2a. Experiments were performed under pulsed quasi-resonant excitation with an effective detuning of 54 meV to the bright exciton. A schematic of the detection concept is presented in Fig. 2b.

**Figure 3:**
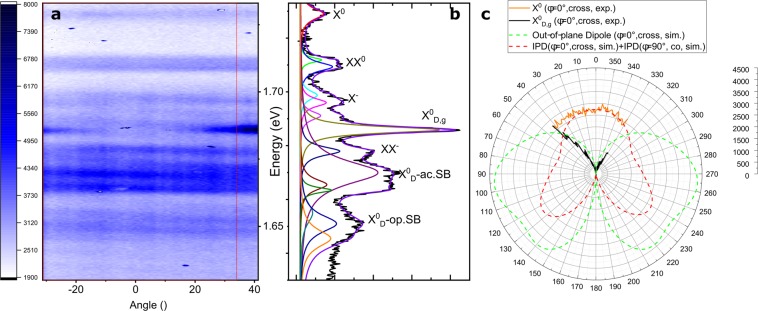
Angle-resolved PL spectrum and emission pattern. (**a**) 2D false-colour contour plot of an angle-resolved PL spectrum of the encapsulated WSe_2_ monolayer under pulsed excitation at 1.789 eV with an excitation density of 78 µJ/cm^2^ at 10 K. (**b**) Corresponding angle-integrated line spectrum (from red-boxed region in a) showing several excitonic features as well as excitonic phonon sidebands. A fit with multiple Lorentzian curves is shown on top of the measured spectrum (dotted curve) with underlying individual peaks labeled (solid curves). (**c**) Comparison of the experimentally obtained radiation patterns for both the bright and the grey exciton to the simulated results. For the in-plane dipole (IPD), the contribution of dipoles perpendicular to the excitation arising from valley dephasing were considered in the simulation, too. Therefore, a degree of linear polarization of 5% was determined from measurements (Fig. SI.3). All intensity scales are linear.

The corresponding two-dimensionally plotted (2D) PL spectrum (energy as a function of emission angle) can be seen in Fig. 3a, which is displayed in false-colour linear intensity scale (white: minimum, dark blue: maximum). Several PL peaks can be identified, which are attributed to a variety of excitonic species, ranging from neutral exciton (X°), charged species (X^−^), biexciton (XX) to grey (X°_D,g_) and dark (X_D_) excitons. Some peaks feature a fine structure, which is taken into account, whereas some peaks arise from acoustic (ac) or optical (op) phonon sidebands (SB), as labeled in the line spectrum in Fig. 3b. For clarity and linewidth analysis, the angle-integrated spectrum has been fitted with a sum of Lorentzian curves (see Fig. 3b), which can be used to describe nearly-homogeneously broadened excitonic lines. For the sake of comparison, all species have been fitted with the same line profile. The obtained line parameters are summarized in Table 1. The peak positions above 1.67 eV are in agreement with Barbone *et al*.^5^ (X°, XX°_1_, XX°_2_, X^−^_inter_, X^−^_intra_, X_D,g_, XX^−^) and Chen *et al.*^6^ (X°, XX°, X^−^_inter_, X^−^_intra_, X_D,g_, XX^−^), while the low energy feature can be explained by the predicted phonon-assisted sideband^13^ emission from the dark excitons (K-K’ and K-Λ transitions). As encapsulation is known to change the band structures as well as the exciton binding energies, above comparison are only done with similarly encapsulated samples. The bright–grey splitting is extracted from the spectrum with Δ = 43 meV. Taking the phonon-band structure from Terrones *et al*.^22^ (with phonon wavenumbers LO 260 cm^−1^, TO 2 cm^−1^, LA 125 cm^−1^ TA 100 cm^−1^) into consideration, the energetic position of the dark exciton arising from the K-K’ transition can be calculated as 1.681 eV and the one from the K-L transition as 1.690 eV, which are in good agreement with predicted values from Brem *et al.*^13^. To further confirm the identification as phonon sidebands, a temperature series has been performed (see Fig. SI.4), the experimental data of which well resembles the prediction of Brem *et al.*^13^.

**Table 1:** Extracted parameters of the PL peaks obtained under quasi-resonant pulsed excitation. A multi-peak fit with Lorentzian line profiles yields the following central energies of different excitonic (X) species and their full width at half maxima (FWHM). The corresponding spectrum with labeled features is shown in Fig. 3b.

Peak	X°	XX°_1_	XX^0^_2_	X^-^_inter_	X^0^ op.SB	X^−^_intra_	X_D,g_	XX^−^	X_D_ (K-K’ TA)	X_D_ (K-K’ LA)	X_D_ (K-Λ TO)	X_D_ (K-Λ LO)	X_D_ (K-K’ TO)	X_D_ (K-K’ LO)
Energy (eV)	1.729	1.712	1.709	1.699	1.696	1.691	1.686	1.678	1.670	1.666	1.663	1.655	1.651	1.646
FWHM (meV)	5.0	2.2	4.0	4.0	3.7	3.9	3.0	4.0	11.0	5.0	2.4	10.0	8.0	8.0

The grey exciton can be easily identified in the angle-dependent spectrum, as it shows a clearly deviating radiation pattern (for a schematic see Fig. 2b). Its position agrees with aforementioned studies^5,6,13,17^. In such 2D spectrum, bright excitons feature an almost constant intensity for all collected angles, while the rather flat profile shows slightly reduced intensities at higher angles. In contrast, the grey exciton is hardly present at 0° emission angle (normal to the sample surface) but exhibits a drastic increase in intensity towards the detection limit of emission angles due to the finite numerical aperture of the objective used in the experiment. A minor tilt in the sample plane with respect to the objective normal even allows us to detect an angle span of approximately +41 to −31°.

To highlight the clear differences in the radiation pattern, the corresponding PL intensity as a function of the emission angle is shown as a polar plot for both the grey and bright neutral exciton in Fig. 3c. The intensity levels are in relation to Fig. 3a and due to a varying background signal level in the range of different densely-packed species, the two species can show different intensity levels at 0°. Astonishingly, one can unambiguously identify the grey exciton due to its expected behavior of radiating in the WSe_2_ plane instead of perpendicular to the monolayer. This does not only provide experimental evidence of such a Γ4 species or give a tool at hand to distinguish them from different excitonic modes, but it also verifies the prediction made by group-theory analysis (cf. Robert *et al.*^15^). In fact, all other excitonic modes such as trions, biexcitons and phonon sidebands show a similar pattern as the representative neutral bright exciton.

From first sight it is clear that the measured patterns do not resemble the well-known dipole radiation pattern. The modification can be a consequence of the surrounding dielectrics and occurring interferences in a multilayer structure. However, in order to verify that the distinct patterns really arise from an in- and out-of-plane dipole, an electromagnetic simulation was done to calculate the farfield pattern. Hereby, the anisotropy of h-BN and WSe_2_ was explicitly taken into account (for further details we refer to the Methods section). Indeed, as can be seen by Fig. 3c, the simulated pattern and the measured pattern are in good agreement with each other. Generally, it can be stated that, for both resonances, the main lobe is surprisingly not mostly directed to the substrate. While being modified by the encapsulation, the difference between the two radiation patterns is still striking. The detailed analysis of the polarization from the simulation can be found in the Supporting Information together with a co- and contra-polarized PL measurement. While the general radiation patterns are not changing, the grey exciton can experimentally only be seen in a cross-polarized measurement, similar to the simulation (cf. Figs. SI.1, SI.2 and SI.3).

## Conclusion

The photoluminescence of h-BN-encapsulated WSe_2_ was analyzed by means of angle-resolved PL spectroscopy. A rich spectrum with numerous excitonic features was obtained that agrees well with previous predictions and measurements on high-quality samples. Strikingly, angle-resolved measurements allow one to clearly distinguish in-plane emitting from out-of-plane emitting excitons, as the analysis of the radiation patterns from excitons shows agreement with electrodynamic simulation. While most of the features show almost no angle dependence, the grey exciton’s signal rises markedly towards higher angles, as predicted for this species. This provides a unique tool for both monolayer samples as well as multilayer stacks, in which various intra- and interlayer excitonic features can form particularly at cryogenic temperatures. This motivates further studies involving charge transfer excitons or hybridized states with partial charge transfer, where a change of dipole moment direction is expected as well.

## Methods

### Sample fabrication

Tungsten diselenide (WSe_2_) bulk single crystals were grown in an excess selenium flux (defect density: 5 × 10^10^/cm^2^). For encapsulated samples, monolayer WSe_2_ and h-BN were first exfoliated from bulk single crystals onto SiO_2_. For WSe_2_, the SiO_2_ substrate was first exposed to an O_2_ plasma step before exfoliation. Monolayers and thin h-BN were both identified by optical contrast using a microscope. Afterwards, a dry stacking technique using polypropylene carbonate (PPC) on PDMS was used to pickup and stack h-BN/WSe_2_ layers. First a top layer of h-BN is picked up at 48 degrees C, then WSe_2_, and finally the bottom layer of h-BN. After each h-BN pickup step the PPC is briefly heated to 90 C to re-smooth the PPC and ensure a clean wave front. For transferring the stack onto a clean substrate (~290-nm SiO_2_ on Si), the substrate is first heated to 75 degrees C, the stack is then put into contact, and gradually heated to 120 degrees C. Afterwards, the PPC/PDMS is lifted and the substrate is immersed in chloroform and rinsed with IPA to remove polymer residue. Atomic-force microscopy confirmed a total stack thickness of ~40 nm (~10 nm + ~30 nm for the encapsulating top and bottom h-BN, respectively).

### PL measurement

The measurements were performed using a conventional 4f µ-PL setup with confocal selection. The sample was mounted in a continuous-flow cryostat at high vacuum and was cooled down to 10 K. A 40x (NA 0.6) microscope objective was used to focus a pulsed Titan-Sapphire laser at 1.789 eV onto the sample. A short-pass filter for 700 nm was used to shape the pulse in front of the sample. A long-pass filter 700 nm and a polarizer after the sample were used to suppress the laser in the collection path of the PL signal, which was detected by a monochromator with nitrogen-cooled camera.

### Simulations

The farfield pattern of the given structure was simulated using CST microwave studio. The thicknesses for the simulation were taken from the AFM measurement of the structure. Furthermore, the anisotropic refractive index of h-BN was taken from Segura *et al.*^23^ and for WSe_2_ a hybrid approach was taken. The out-of-plane refractive index was taken from Laturia *et al.*^24^, ${{\epsilon }}_{\infty ,z}={{\epsilon }}_{s,z}=7.5$. For the in-plane permittivity, a Lorentz model was employed to account for the resonance of the bright A-1s transition. Here, the following values were used: ${{\epsilon }}_{\infty ,x,y}=15,\,{{\epsilon }}_{s,xy}=15.22$ and a damping frequency of 4.77 THz. The contribution of the excitons, especially the grey exciton, to the permittivity in the out-of-plane direction is about 1000 times weaker than for the in-plane component^17,25^, giving no significant contribution to the permittivity. Therefore, this contribution was neglected for the simulation. For silicon oxide and silicon, they were taken from the programs database. The resulting farfield patterns were analyzed in terms of polarization by projecting them on the unit vector of the radiation sphere using the Ludwig 3 convention.

### Visualization

The schematic depiction of the WSe_2_ monolayer in Fig. 1b is based on crystallographic data provided by the *Materials Project*^26^ and drawn by the tool *Mercury*^27^.

## Supplementary information

Supplementary Information.
