# Supplementary material for: Direct Measurement of the Radiative Pattern of Bright and Dark Excitons and Exciton Complexes in Encapsulated Tungsten Diselenide
Source: Sci Rep. 2020 May 15;10:8091. doi: 10.1038/s41598-020-64838-z (PMC7229226; doi:10.1038/s41598-020-64838-z)
Supplement: Supplementary file 1 — Supplementary Information. [file 41598_2020_64838_MOESM1_ESM.pdf]

Supporting Information

Direct Measurement of the Radiative Pattern of Bright and Dark Excitons and Exciton Complexes in Encapsulated Tungsten Diselenide

Lorenz Maximilian Schneider[1,*], Shanece S. Esdaille[2], Daniel A. Rhodes[2], Katayun Barmak[3], James C. Hone[2], and Arash Rahimi-Iman[1,*]

[1]*Faculty of Physics and Materials Sciences Center, Philipps-Universität Marburg, Marburg, 35032, Germany*

[2]*Department of Mechanical Engineering, Columbia University, New York, NY 10027, USA*

[3]*Department of Applied Physics and Applied Mathematics, Columbia University, New York, NY 10027, USA*

S1. Polarization analysis

The simulation was once performed for an in-plane and out-of-plane dipole located in the middle of the monolayer, respectively. The far-field patterns were calculated at different spectral positions, i.e. that of the bright and the grey exciton, as displayed in. **Figure SI.1**. One can see that for this structure, the patterns are indeed modified; nevertheless, the emission from in- and out-of-plane dipoles can be still easily distinguished. While the in-plane dipole has a well-defined orientation in the simulation, in experiment data the emission is expected to be found for a preferred direction of microscopic dipoles (i.e. along the lasers polarization), but not exclusively for that direction owing to valley dephasing. This is especially important for the here used rather strong pulsed excitation, which favours these dephasing processes.

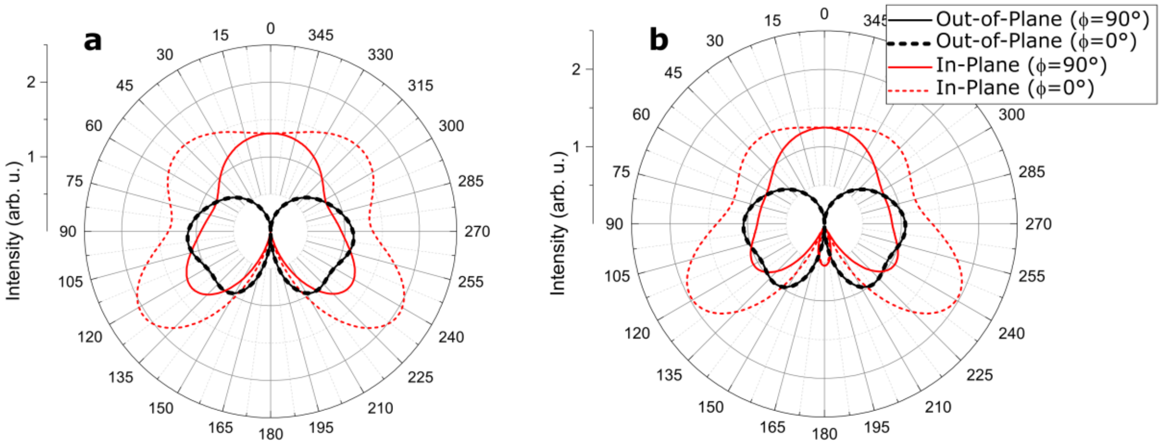

Figure SI.1 | Simulated radiation patterns. Farfield intensity patterns simulated for an electronic dipole emission at the spectral position of the bright exciton (a) as well as the grey/dark exciton (b) for both linear polarizations. Both polar diagrams show the radiation patterns for an in-plane (located along the x-axis) and an out-of-plane dipole, in the x-z (ϕ=0°, dashed line) and y-z (90°, solid line) planes. The pattern for the out-of-plane dipole has been multiplied by 100 to show all data on the same intensity scale, whereas the profiles for 0 and 90° are congruent. The 90-270° line corresponds to the monolayer orientation, with angles between those values representing emission to the substrate side.

This means that a weighted average over both cases, i.e. parallel and perpendicular to the dipole, is expected in experiment. For the out-of-plane dipole, however, we find no dependence of the emission pattern to the selected plane (x-z or y-z).

In a next step, the polarization was evaluated by projecting the polarization of the beams emitted from a dipole to the unit-vectors of a sphere according to the Ludwig 3 convention for the relevant cases (see **Figure SI.2**). For the in-plane dipole, the simulation shows that the pattern indeed varies with the polarization, i.e. detection co- (black) or cross-polarization (red). Nevertheless, as the difference in magnitude between co- and cross polarization is strong (from 2.5 to 8 orders of magnitude), the contribution of dipoles oriented 90° with respect to the laser polarization arising from valley decoherence will be more significant. For the out-of-plane dipole, it is remarkable that for a fixed detection plane, e.g. for ϕ=0°, the radiation is only found for cross-polarized emission. Orientations with negligible value level (<<1E-5) are neglected in the polar plots.

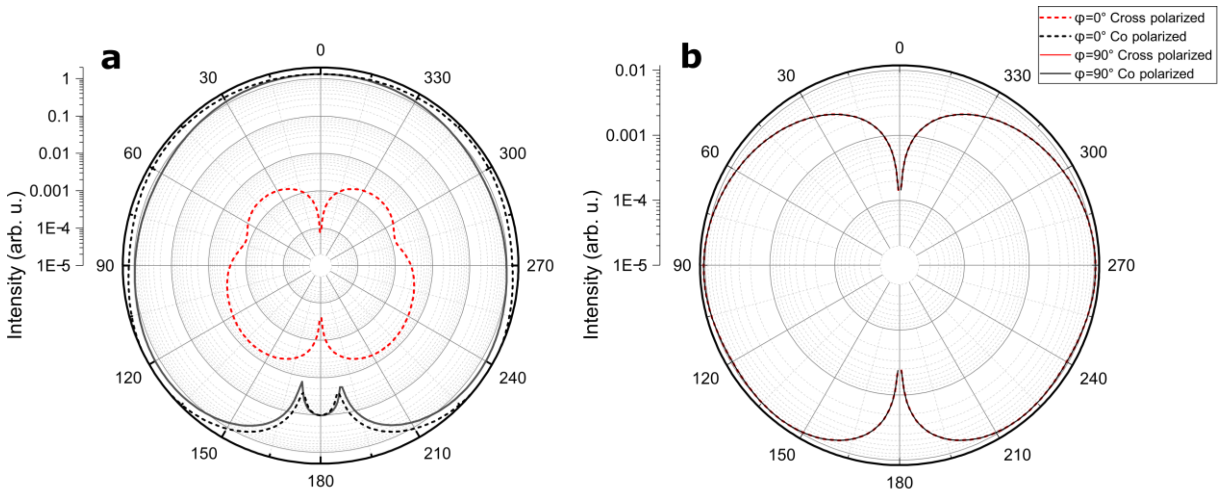

Figure SI.2 | Analysis of the polarization for the simulation results. Intensity profile of detected signal for co-/cross-polarized light in the plane parallel (ϕ=0°), as well as perpendicular (ϕ=90°), to the dipole. The analysis has been performed for an in-plane dipole at the spectral position of the bright exciton (a), as well as for an out-of-plane dipole at that of the grey exciton (b).

Furthermore, to check the polarization dependence of the emission pattern experimentally, an angle-resolved measurement has been performed under linearly co- and cross-polarized detection (see **Figure SI.3**).

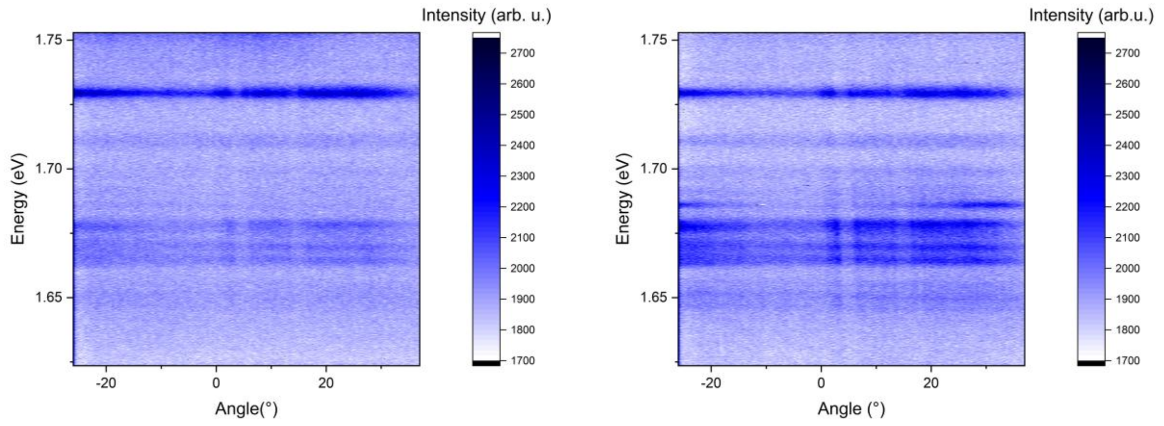

Figure SI.3 | Co- and cross-polarized emission spectra. Angle-resolved measurements of photoluminescence under co- and cross-polarization (left and right, respectively) in the plane parallel to the laser excitation (ϕ=0°).

The most remarkable difference between these measurements is the absence of the grey exciton under co-polarized emission, which is in good agreement with simulated predictions for the selected detection plane.

S2. Temperature Series

A temperature series performed on a high-quality hBN/WSe2/hBN stack on a SiO2/Si substrate verifies the presence of phonon side bands (see **Figure SI.4**). A comparison to theoretical predictions of Ref. [13] reveals that the experimental temperature dependence of the identified phonon sidebands is in good agreement with calculations, as the contour diagram with normalized spectra and relative energies in **Figure SI.4** shows.

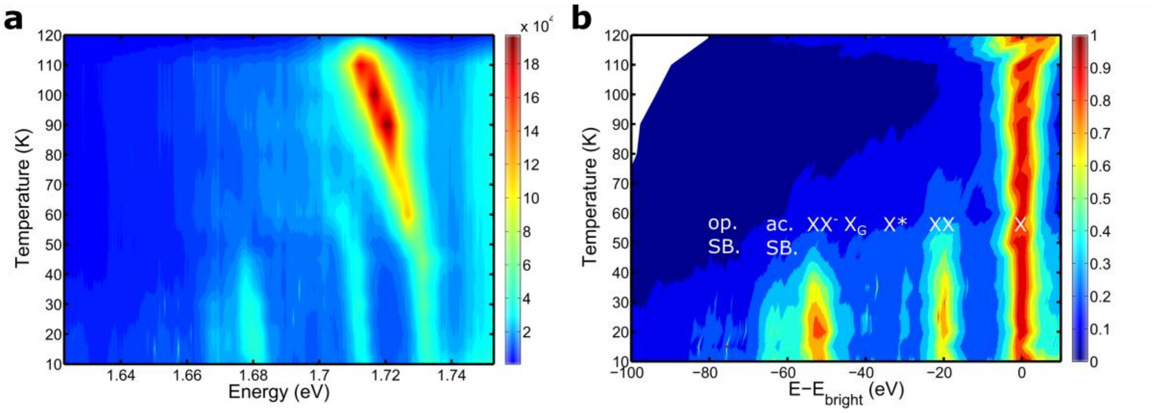

Figure SI.4 | Temperature-dependent photoluminescence. Raw µ-PL data of the temperature series presented in contour diagram stitched from individual line spectra (a). Temperature-dependent spectra normalized and plotted relative to the exciton energy (b). Here, both the representation as well as the signatures' behavior is similar to that in the literature concerning theoretical predictions[13] (not shown here).
